# Imaging angiogenesis in atherosclerosis in large arteries with ^68^Ga-NODAGA-RGD PET/CT: relationship with clinical atherosclerotic cardiovascular disease

**DOI:** 10.1186/s13550-021-00815-5

**Published:** 2021-08-14

**Authors:** Matthieu Dietz, Christel H. Kamani, Emmanuel Deshayes, Vincent Dunet, Periklis Mitsakis, George Coukos, Marie Nicod Lalonde, Niklaus Schaefer, John O. Prior

**Affiliations:** 1 Nuclear Medicine and Molecular Imaging Department, Lausanne University Hospital, Rue du Bugnon 46, 1011 Lausanne, Switzerland; 2 Nuclear Medicine Department, Montpellier Cancer Institute (ICM), University of Montpellier, 208 Avenue des Apothicaires, 34298 Montpellier Cedex 5, France; 3 Department of Diagnostic and Interventional Radiology, Lausanne University Hospital, Rue du Bugnon 46, 1011 Lausanne, Switzerland; 4 Ludwig Institute for Cancer Research and Department of Oncology, Lausanne University Hospital, Rue du Bugnon 46, 1011 Lausanne, Switzerland

**Keywords:** Atherosclerosis, Angiogenesis, ^68^Ga-NODAGA-RGD, PET/CT

## Abstract

**Background:**

Integrin alpha-V-beta-3 (αvβ3) pathway is involved in intraplaque angiogenesis and inflammation and represents a promising target for molecular imaging in cardiovascular diseases such as atherosclerosis. The aim of this study was to assess the clinical correlates of arterial wall accumulation of ^68^Ga-NODAGA-RGD, a specific α_v_β_3_ integrin ligand for PET.

**Materials and methods:**

The data of 44 patients who underwent ^68^Ga-NODAGA-RGD PET/CT scans were retrospectively analyzed. Tracer accumulation in the vessel wall of major arteries was analyzed semi-quantitatively by blood-pool-corrected target-to-background ratios. Tracer uptake was compared with clinically documented atherosclerotic cardiovascular disease, cardiovascular risk factors and calcified plaque burden. Data were compared using the Mann–Whitney *U* test, Pearson correlation and Spearman correlation.

**Results:**

^68^Ga-NODAGA-RGD arterial uptake was significantly higher in patients with previous clinically documented atherosclerotic cardiovascular disease (mean TBR 2.44 [2.03–2.55] vs. 1.81 [1.56–1.96], *p* = 0.001) and showed a significant correlation with prior cardiovascular or cerebrovascular event (*r* = 0.33, *p* = 0.027), BMI (*ρ* = 0.38, *p* = 0.01), plaque burden (*ρ* = 0.31, *p* = 0.04) and hypercholesterolemia (*r* = 0.31, *p* = 0.04).

**Conclusions:**

^68^Ga-NODAGA-RGD holds promise as a non-invasive marker of disease activity in atherosclerosis, providing information about intraplaque angiogenesis.

## Introduction

Cardiovascular atherosclerotic disease is the leading cause of death worldwide [1]. Atherosclerosis is a systemic condition consisting of the accumulation of fatty and/or fibrous material in the subendothelial space (intima) of medium- and large-sized arteries. This process results in the formation of progressive inflammatory plaques that represent the hallmark lesion [2]. The development of atherosclerosis is an intricate process of cellular alterations over a prolonged period. In atherosclerotic lesions, the combination of macrophage infiltration and apoptotic death together with hypoxia-induced necrosis is thought to promote neovascularization [3]. Angiogenesis within the vessel wall is comprised of a network of capillaries that arise from the adventitial vasa vasorum and extend into the intimal layer. These capillaries are thought to be important regulators of plaque growth and as a key factor in lesion instability. Increased density of capillaries is associated with intraplaque hemorrhage and plaque rupture [3, 4].

Combined positron emission tomography and computed tomography (PET/CT) is a non-invasive hybrid imaging technique that could potentially be used to measure inflammatory activity within the vasculature, the most numerous publications being with ^18^F-FDG [5]. However, a more straightforward marker of atherosclerosis, with less physiologic uptake, no interaction with blood glucose and no requirement for a fasting period before imaging could be of interest. Integrins αvβ3 are transmembrane glycoproteins that are involved in the migration of activated endothelial cells during the formation of new vessels. Integrins αvβ3 are expressed in endothelial cells, medial and some intimal smooth muscle cells. Expression of αvβ3 integrin was also found in CD68-positive macrophages in the shoulder of advanced plaques and in the perimeter of the necrotic core of atherosclerotic lesions [6]. Integrin αvβ3 contains a distinctive RGD-amino acid sequence (arginine-glycine-aspartate) in the cell-ligand interaction site. Hence, a few RGD-based PET agents have been tested for imaging integrin in atherosclerosis, mainly in preclinical models [7–11], and in two recent clinical evaluations [12, 13].

^68^Ga-NODAGA-RGD is an emerging RGD-based PET radiotracer with strong affinity for the αvβ3 integrin [14, 15]. We hypothesized that ^68^Ga-NODAGA-RGD may act as an imaging marker of inflammation and angiogenesis in atherosclerosis. The purpose of this study was to examine the relationship between arterial wall ^68^Ga-NODAGA-RGD uptake in large arteries and the incidence of atherosclerotic cardiovascular diseases.

## Material and methods

### Patients

The population of this retrospective study consisted of consecutive patients who had been referred to our institution for a ^68^Ga-NODAGA-RGD PET/CT within clinical study protocols. Included in this analysis were trials assessing tumoral angiogenesis (NCT02666547, NCT03475134), cardiac lesions angiogenesis (NCT03809689) and inflammatory atheromatous plaques in the carotid arteries (NCT01608516) [15]. Patients were included if they underwent a ^68^Ga-NODAGA-RGD PET/CT from vertex to mid-thigh. Patients could not be included if they did explicitly refuse the retrospective use of their data for research. All procedures performed in this study were in accordance with the ethical standards of the institutional and/or national research committee and with the 1964 Helsinki declaration and its last amendments or comparable ethical standards. The Ethics Committee Vaud (CER-VD) approved this retrospective study protocol (CER-VD #2018_01513) and waived the need for patient informed consent for the study analysis.

### Clinical data

Clinical data were collected retrospectively from the medical records of the patients. History of atherosclerotic cardiovascular disease (ASCVD) was collected, according to the strict same definitions as listed by Mach et al. [16], including previous acute coronary syndrome (myocardial infarction or unstable angina), stable angina, coronary revascularization (percutaneous coronary intervention, coronary artery bypass graft surgery and other arterial revascularization procedures), stroke and transient ischemic attack, and peripheral arterial disease. Subjects with any type of clinically documented ASCVD were classified into the ASCVD group. Subjects with no clinically documented ASCVD were classified into the control group. Cardiovascular risk factors including age, sex, body mass index (BMI), arterial hypertension, hypercholesterolemia, smoking (current or former) and diabetes mellitus were also collected for every subject. Potential last previous systemic anticancer therapies and time intervals between last systemic anticancer therapies and PET scans were also collected. Because of the potential influence of statins on expression of vascular endothelial growth factor on monocytes, treatments with statins were also recorded [17].

### Image acquisitions

^68^Ga-NODAGA-RGD PET/CT were performed at our hospital. Pregnancy was excluded in women of childbearing age before each PET/CT. ^68^Ga-NODAGA-RGD PET/CT images were acquired 63 [59–71] minutes after intravenous administration of 190 [175–210] MBq of ^68^Ga-NODAGA-RGD in an antecubital vein followed by 10 mL of 0.9% NaCl solution.

Images were acquired on a Discovery 690 TOF (GE Healthcare, Waukesha, WI, USA), or a Biograph Vision 600 (Siemens Medical Solutions, Knoxville, USA) PET/CT. Acquisitions were performed with 3 min per bed position (Discovery), or a continuous flow mode (Biograph Vision). PET data were reconstructed using OSEM (Discovery: 3 iterations, 16 subsets; Biograph Vision: 4 iterations, 5 subsets). Head to mid-thigh unenhanced CT was acquired for attenuation correction (Discovery: 120 kV, 40 mA, 0.8 s/rotation, pitch 0.9; Biograph Vision: 100 kV, 40 mA, 0.5 s/rotation, pitch 0.8).

### Image analysis

Transaxial PET, CT, and fused ^68^Ga-NODAGA-RGD PET/CT images were analyzed both visually and semi-quantitatively on a dedicated workstation (Syngo.Via, VB30 Siemens Healthcare), blinded to the patient’s clinical information.

### ^***68***^***Ga-NODAGA-RGD uptake***

Maximal standardized uptake values (SUVmax) for ^68^Ga-NODAGA-RGD were measured in the following arterial segments: both common carotid arteries, ascending aorta, aortic arch, descending aorta, abdominal aorta and both iliac arteries, using a previously validated method [18]. Briefly, on axial coregistered PET/CT slices, simple circular regions of interest (ROIs) were placed to cover arterial walls and lumen. For those vessels of greater diameter, 1-cm-diameter ROIs were placed along the arterial walls and were slid along the arterial segments to locate the highest SUVmax within the tubular arterial segment, avoiding areas of ^68^Ga-NODAGA-RGD spillover. For blood pool SUV measurements, three different 1-cm-diameter ROIs were placed in both the mid-lumen of the inferior and superior vena cava, and the SUVmean of the six measurements was collected (blood pool activity = average of the six SUVmean measurements). SUVmax were then corrected for blood pool activity to provide tissue-to-background ratios (TBR = SUVmax/blood pool activity) measurements, as a measure of arterial tracer uptake [18]. The average TBR (mean TBR) was calculated for each patient, considering all assessed segments. TBRaorta was the average of ascending aorta, aortic arch, descending aorta and abdominal aorta TBR measurements. TBRcarotid was the average of both common carotid arteries TBR measurements. TBRiliac was the average of both iliac arteries TBR measurements.

### Plaque burden

The CT scans were examined for the presence of calcified plaque (high-density mural areas with attenuation > 130 Hounsfield units) in the walls of the same arterial segments investigated by PET [19]. The amount of calcification was semi-quantitatively ranked according to a previously validated scale [18]:

- 0: absent calcified plaque,
- 1: small, calcified plaque covering less than 10% of the vessel circumference,
- 2: calcified plaque involving 10–25% of the vessel circumference,
- 3: calcified plaque involving 25–50% of the circumference,
- 4: calcified plaque involving more than 50% of the vessel circumference.

The calcified plaque scores were then summed for the eight areas [18].

### ***Direct visual comparisons of ***^***68***^***Ga-NODAGA-RGD foci on PET/CT images to calcification sites on CT images***

To understand if ^68^Ga-NODAGA-RGD foci are located or not in calcified plaques on CT images, visual comparisons of highest ^68^Ga-NODAGA-RGD foci to calcification sites on CT were evaluated within the fifty tubular arterial segments with the highest TBR measurements.

### Statistics

We assessed the distribution of data with the Shapiro–Wilk test. Continuous parametric variables were expressed as mean ± SD and compared using Student’s *t* tests. Nonparametric data were presented as median [interquartile range] and compared using the Mann–Whitney *U* test. Categorical variables were collected as numbers (*n*) and percentages (%) and compared using the chi-square test or Fisher exact test. Pearson correlation analysis (*r*) or Spearman correlation analysis (*ρ*) was used to correlate mean TBR with prior cardiovascular and cerebrovascular event, age, sex, BMI, arterial hypertension, hypercholesterolemia, smoking and diabetes mellitus. A *p* value less than or equal to 0.05 was considered statistically significant. The statistical analysis was performed using R version 4.0.3 (R Foundation for Statistical Computing, Vienna, Austria).

## Results

### Patients

**Fig. 1:**
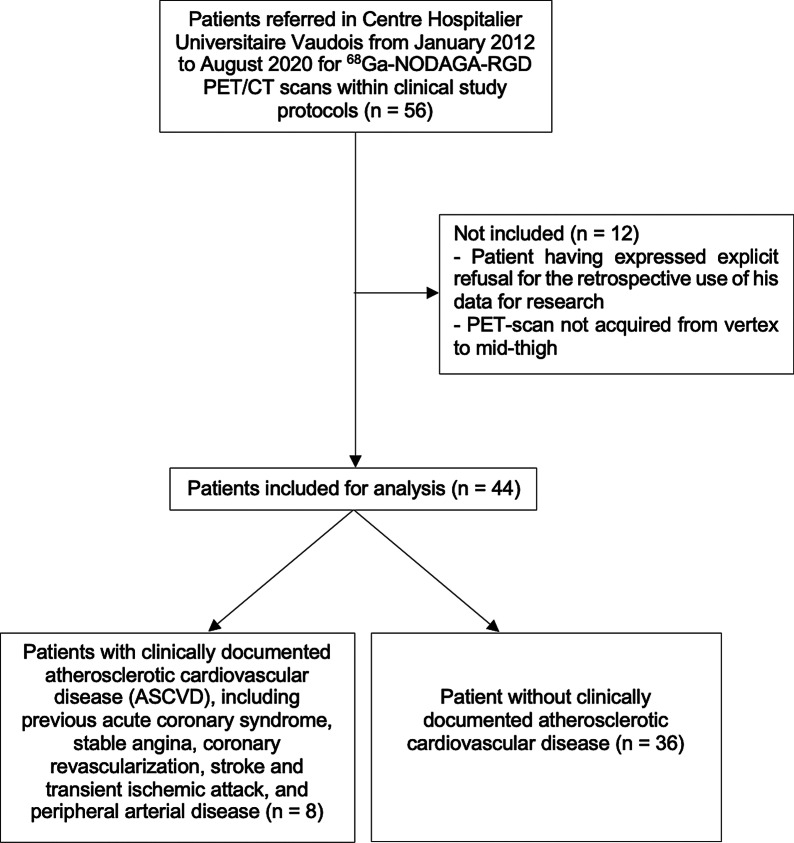
Flowchart

In total, fifty-six patients underwent a ^68^Ga-NODAGA-RGD PET/CT at our institution, and forty-four patients could be included retrospectively. A flowchart of the study design is shown in Fig. 1. Clinical characteristics of the patients are reported in Table 1. Thirty-nine of forty-four patients (89%) were referred within oncologic studies and five of forty-four patients (11%) were referred before carotid endarterectomy.

**Table 1:** Patient characteristics

Characteristics	Data
Patients (n)	44
Sex (n)	
Men	24 (55%)
Women	20 (45%)
Age (y)	60 [53–66]
Body mass index (kg/m^2^)	28 ± 4
Previous clinically documented ASCVD*	
Coronary revascularization^†^	6 (14%)
Myocardial infarction	5 (11%)
Stroke and transient ischemic attack	2 (5%)
Peripheral arterial disease	2 (5%)
Statin therapy (n)	8 (18%)
Acetylsalicylic acid treatment	10 (23%)
Systemic inflammatory disease	
Rheumatoid arthritis	1 (2%)
Ulcerous-hemorrhagic recto-colitis	1 (2%)
Cardiovascular risk factors (n)	
Arterial hypertension	13 (30%)
Hypercholesterolemia	12 (27%)
Smoking (current or former)	19 (43%)
Diabetes mellitus	4 (9%)
Type of disease (n)	
Head and neck tumors	10 (23%)
Melanoma	10 (23%)
Esophagus carcinoma	6 (14%)
Carotid endarterectomy	5 (11%)
Lung carcinoma	2 (5%)
B-cell lymphoma	2 (5%)
Ovarian cancer	2 (5%)
Pancreatic cancer	2 (5%)
Stomach cancer	2 (5%)
Leiomyosarcoma	1 (2%)
Breast cancer	1 (2%)
Glioma	1 (2%)

*ASCVD: atherosclerotic cardiovascular disease

^†^Percutaneous coronary intervention or coronary artery bypass graft surgery

Eight of the forty-four patients (18%) had previous myocardial infarction, coronary revascularization, stroke or transient ischemic attack, and/or peripheral arterial disease and were thus classified into the ASCVD group. The thirty-six remaining patients (82%) were classified into the control group. All the five patients referred for carotid endarterectomy were included in the ASCVD group. In this group, the three remaining patients were referred for esophagus, lung, and head and neck tumors. The median age was higher in the ASCVD than in the control group, and the ASCVD group included a higher proportion of men as compared to the control group (respectively, 64 years [61–72] vs. 59 years [50–65], *p* = 0.059, and 7 men of 8 patients; 87%, vs. 17 men of 36 patients; 46%, *p* = 0.054). The ASCVD group included a higher prevalence of hypertension (5 of 8 patients; 62%) as well of hypercholesterolemia (6 of 8 patients; 75%), as compared to the control group (respectively, 8 of 36 patients; 23%, *p* = 0.037, and 6 of 36 patients; 17%, *p* = 0.003). BMI, smoking and diabetes mellitus did not significantly differ between the two groups.

All the 39 oncology patients were referred for baseline PET/CT before receiving additional treatments (chemotherapy, anti-angiogenic therapy or surgery). Among them, 14 patients had previous systemic anticancer therapies, with a median time interval of 113 [22–189] days between last systemic anticancer therapies and PET scans. The last previous systemic anticancer therapy for each of these 14 patients and time intervals between last systemic anticancer therapies and PET scans are shown in Table 2. None of these last previous systemic anticancer therapies included antiangiogenic drugs. Six patients had systemic anticancer therapy within 4 weeks before imaging.

**Table 2:** Description of the last previous systemic anticancer therapy and the time interval between the last systemic anticancer therapy and the PET scan for each of the 14 concerned patients

Patient	Last previous systemic anticancer therapy	
	Type of therapy	Days before PET scan
1	Trastuzumab, cisplatine pertuzumab	127
2	Cyclophosphamide, oxaliplatine, adriamycin	288
3	Carbotaxol	100
4	Nab-paclitaxel	9
5	Cobimetinib, vemurafenib	28
6	Dabrafenib, trametinib	16
7	Cisplatin, vindesine	20
8	Nivolumab, ipilimumab	208
9	Nivolumab	131
10	Spartalizumab	321
11	Pembrolizumab	130
12	Nivolumab, ipilimumab	46
13	Encorafenib binimetinib	1
14	Pembrolizumab	378

When the mean TBR values were compared, there was no difference between patients who had previous systemic anticancer therapies and patients who had not previous systemic anticancer therapies (1.84 [1.57–1.89] vs. 1.89 [1.62–2.24], respectively, *p* = 0.33).

### ***Arterial Wall ***^***68***^***Ga-NODAGA-RGD uptake and plaque burden***

In the entire group of 44 patients, a total of 352 arterial segments were evaluated and statistically analyzed. The PET/CT was acquired on the Discovery PET/CT in 28 patients and on the Biograph Vision PET/CT in the remaining 16 patients. When blood pool activities were compared, no significant difference was found between Discovery PET/CT and Biograph Vision PET/CT acquisitions (1.03 [0.87–1.18] g/mL vs. 1.11 [0.91–1.23] g/mL, respectively, *p* = 0.25). Furthermore, when the mean TBR values were compared, there was no difference between patients who had Discovery PET and patients who had Biograph Vision PET (1.77 [1.56–2.10] vs. 1.89 [1.81–2.03], respectively, *p* = 0.37).

**Fig. 2:**
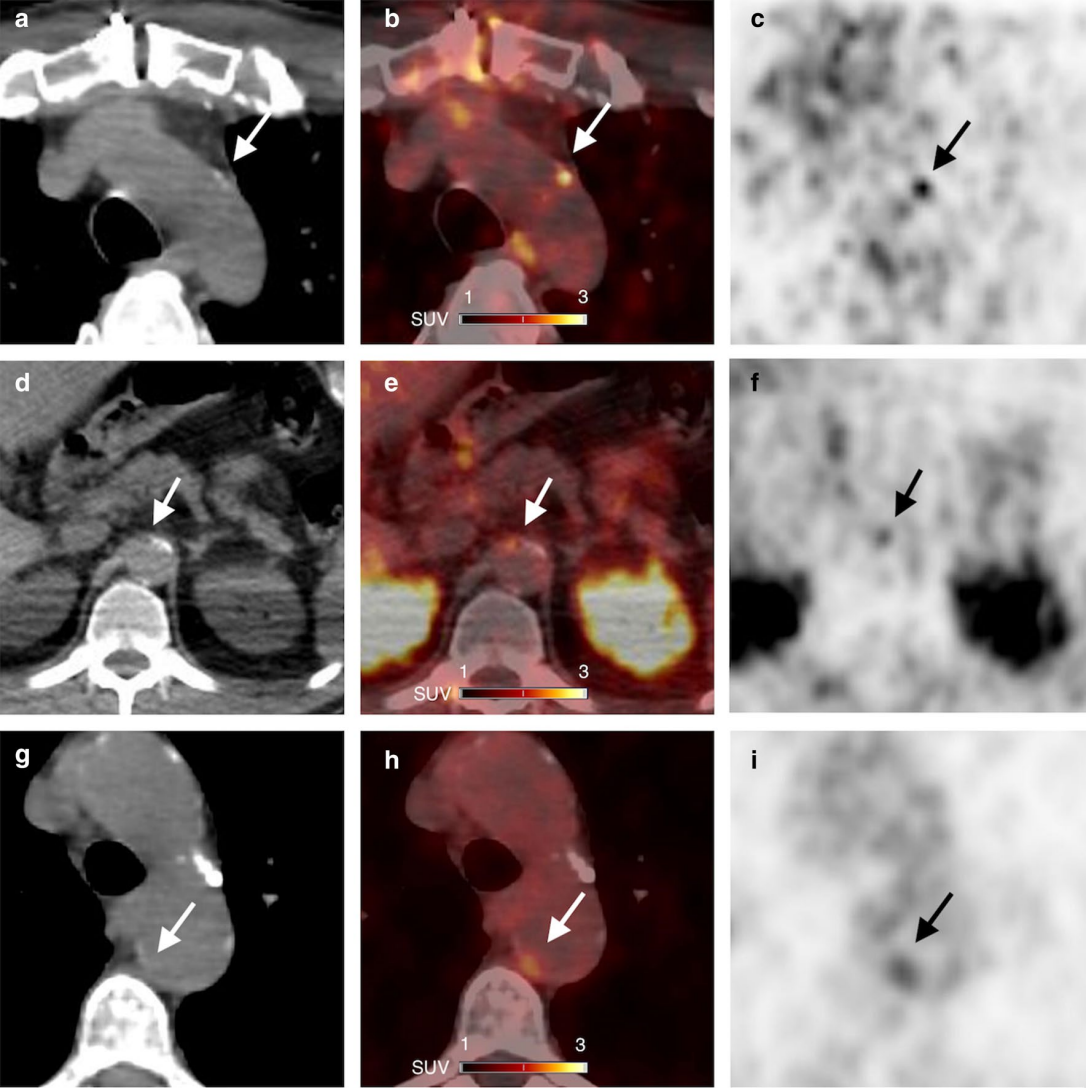
CT (**A**, **D**, **G**), PET/CT (**B**, **E**, **H**) and PET images (**C**, **F**, **I**) in axial views show foci of ^68^Ga-NODAGA-RGD arterial uptake in partly calcified atherosclerotic lesions (arrows): (**A**, **B**, **C**) in wall of arch aorta in a 62-year-old man who had a myocardial infarction 5 months before PET imaging; (**D**, **E**, **F**) in wall of abdominal aorta in a 54-year-old man who had a transient ischemic attack one week before PET imaging; (**G**, **H**, **I**) in wall of arch aorta in a 74-year-old woman who had a myocardial infarction 33 months before PET imaging

The highest TBRs were documented in the descending and abdominal aorta (1.97 [1.72–2.33] and 2.09 [1.69–2.57], respectively), whereas the lowest TBRs were seen in the ascending aorta and common carotid arteries (1.67 [1.39–2.05] and 1.57 [1.31–1.78], respectively). Among the fifty tubular arterial segments with the highest TBR measurements, 13 (26%) highest ^68^Ga-NODAGA-RGD foci did correspond to CT calcification, and 37 (74%) foci did not correspond to CT calcification. Examples of foci of ^68^Ga-NODAGA-RGD arterial uptake are shown in Fig. 2. Calcified plaque scores were the highest in the abdominal aorta (2 [0–4]).

### Clinical baseline characteristics and plaque burden correlation

**Fig. 3:**
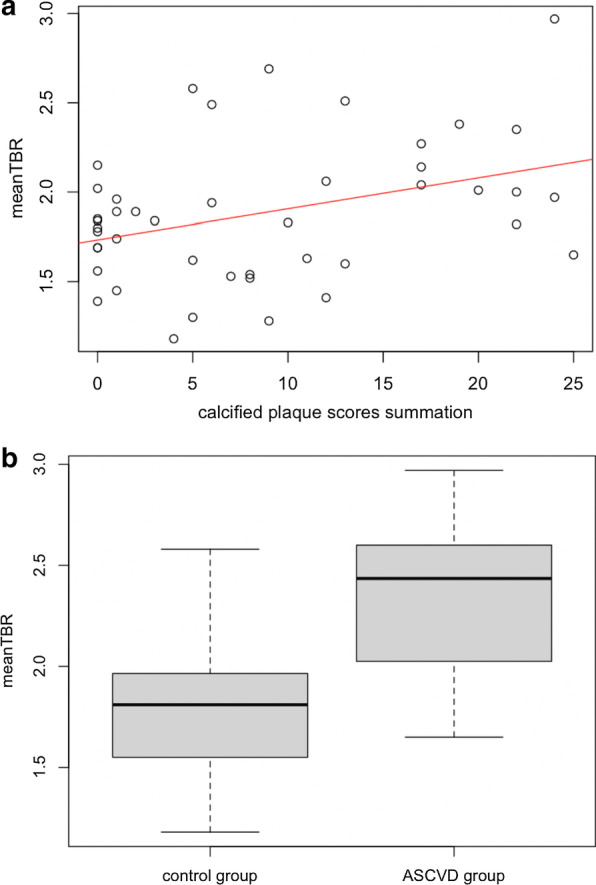
**A** Scatterplot showing correlation of mean TBR of ^68^Ga-NODAGA-RGD uptake and calcified plaque scores summation of eight arterial segments on per-patient basis. **B** Box plot showing mean TBR for all patients, separated into those with clinically documented atherosclerotic cardiovascular disease (ASCVD group), and those without clinically documented atherosclerotic cardiovascular disease (control group)

**Fig. 4:**
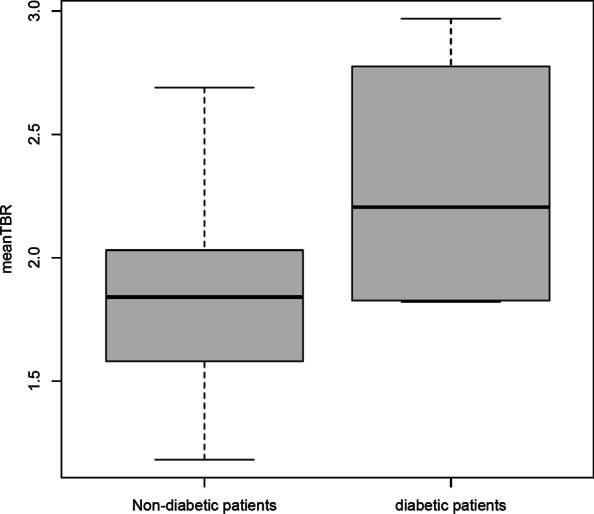
Box plot showing mean TBR for all patients, separated into non-diabetic patients and diabetic patients

Prior cardiovascular or cerebrovascular event significantly correlated with ^68^Ga-NODAGA-RGD mean TBR (*r* = 0.33, *p* = 0.027). ^68^Ga-NODAGA-RGD mean TBR also showed a significant correlation with BMI (*ρ* = 0.38, *p* = 0.01), plaque burden (*ρ* = 0.31, *p* = 0.04; Fig. 3) and hypercholesterolemia (*r* = 0.31, *p* = 0.04). There was no significant correlation between ^68^Ga-NODAGA-RGD mean TBR and smoking, age, diabetes mellitus and arterial hypertension. Interestingly, ^68^Ga-NODAGA-RGD mean TBR showed nevertheless a tendency for a higher uptake in diabetic patients (2.21 [1.83–2.68] in diabetic patients vs. 1.84 [1.54–2.02] in non-diabetic patients, *p* = 0.14; Fig. 4).

### Quantitative assessment across groups

Quantitative assessment is reported in Table 3. With blood-pool activities being comparable between both groups (0.98 [0.89–1.06] g/mL in the ASCVD group vs. 1.07 [0.89–1.27] g/mL in the control group, *p* = 0.39), mean TBR (Fig. 3) and TBRaorta of ^68^Ga-NODAGA-RGD were significantly higher in the ASCVD group as compared to the control group (respectively, 2.44 [2.03–2.55] vs. 1.81 [1.56–1.96], *p* = 0.001; and 2.51 [2.31–2.81] vs. 1.86 [1.62–2.04], *p* = 0.001). TBRcarotid and TBRiliac showed a tendency for a higher uptake in the ASCVD group (respectively, 1.69 [1.66–2.26] vs. 1.49 [1.27–1.77], *p* = 0.08; and 2.43 [1.68–2.93] vs. 1.92 [1.55–2.14], *p* = 0.098).

**Table 3:** Quantitative assessment per group

Characteristic	ASCVD^§^ group	Control group	*p* Between groups
Patients (*n*)	8	36	
Mean TBR	2.44 [2.03–2.55]	1.81 [1.56–1.96]	0.001
TBR_aorta_	2.51 [2.31–2.81]	1.86 [1.62–2.04]	0.001
TBR_carotid_	1.69 [1.66–2.26]	1.49 [1.27–1.77]	0.08
TBR_iliac_	2.43 [1.68–2.93]	1.92 [1.55–2.14]	0.098
Blood pool activity (g/mL)	0.98 [0.89–1.06]	1.07 [0.89–1.27]	0.39

^§^*ASCVD* atherosclerotic cardiovascular disease

## Discussion

Our main results are that ^68^Ga-NODAGA-RGD arterial wall uptake was higher in patients with previous clinically documented ASCVD and correlated with prior cardiovascular or cerebrovascular event and with progressive atherosclerotic plaque burden. Our study would suggest that ^68^Ga-NODAGA-RGD holds promise as a non-invasive marker of disease activity in atherosclerosis, providing information on key features of high-risk atheroma: inflammation and angiogenesis.

Both inflammation and angiogenesis processes are associated with atheroma progression, plaque rupture and clinical events. The necrotic core in culprit plaques forms as result of increasing inflammation [20]. In response to pro-atherogenic stimuli, activated monocytes infiltrated within the intima and differentiate into pro-inflammatory macrophages [21]. While progressing, atherosclerotic plaques will develop a lipid-rich or necrotic core, resulting from the apoptosis of the resident pro-inflammatory macrophages [20]. Pro-inflammatory macrophage activities within the atherosclerotic plaque lead to the weakening of the protective fibrous cap, mediated by the matrix metalloproteinases, that degrade the extracellular matrix components, predisposing it to rupture [22]. Angiogenesis is believed to occur in response to hypoxic conditions within the necrotic core. Indeed, increasing wall thickness during atherosclerosis leads to a reduction of the intravascular oxygen amount, a situation further exacerbated by the increased oxygen consumption of high metabolic activated inflammatory cells within the atherosclerotic plaque [23]. Vascular endothelial growth factor further modulates the activation state of the adventitial vasa vasorum endothelial cells to a highly migratory and proliferative state, resulting in neovessels formation toward the base of the plaque [24]. Neovessels, arising from the adventitial vasa vasorum, grow into the base of progressive atherosclerotic lesions and provide an alternative entry pathway for monocytes and immune cells. The plaque neovessels are fragile and leaky, giving rise to local extravasation of plasma proteins and erythrocytes [25]. Plaque hemorrhage itself results in a pro-inflammatory response, plaque destabilization and clinical events [3, 4].

The use of the PET technique to visualize inflammation in vivo in atherosclerosis in large arteries has been performed with success using tracers such as ^18^F-FDG, DOTA-derived somatostatin analogs or ^68^Ga-Pentixafor [18, 26–28]. A non-invasive imaging technique that can inform about the activity of two adverse pathological processes, namely inflammation and angiogenesis, might therefore be even more accurate in identifying patients with active high-risk atheroma and potentially predicting risk of rupture. Hence, over the past decade, pre-clinical studies using RGD-based tracers have shown interesting results. In vivo imaging with a small animal PET/CT demonstrated ^18^F-galacto-RGD PET signal corresponding to the advanced calcified plaques of the aortic arch region of hypercholesterolemic mice [11]. Another study on atherosclerotic mice showed accumulation of ^68^Ga-DOTA-RGD into aortic plaques [9]. The role of a single photon emission computed tomography ^99m^Tc-RGD-based probe in detection of inflammation in mouse models of carotid arteries remodeling has been demonstrated [8]. More recently, Su et al. demonstrated a ^18^F-labeled RGD preferentially binds to aortic plaque in an ApoE knock out mouse model of atherosclerosis, and Golestani et al. demonstrated a good correlation between ^18^F-RGD-K5 uptake and intraplaque neovessels density in carotid endarterectomy specimens [7, 10].

The discussion about the assessment of the atherosclerotic inflammatory activity as a marker of plaque vulnerability relies among others on data showing that non-obstructive coronary artery disease is responsible for most acute coronary syndromes [29, 30]. Moreover, some data from catheterization laboratories have shown a high proportion of significant stenosis (> 70% reduction of the coronary lumen) of culprit lesions in patients presenting with ST-segment elevation myocardial infarction [31]. In the same line, it has been demonstrated that acute coronary event resulting from obstructive coronary plaque with prior inducible ischemia has better outcome in comparison with acute coronary event from non-obstructive coronary plaque without prior inducible ischemia [32]. The protective adaptative changes resulting from the myocardial preconditioning could explain these different outcomes depending on the severity of the artery lumen stenosis and the presence of inducible ischemia prior acute coronary event [33]. Nevertheless, these observations further confirm that other criteria above the solely angiographic evaluation of the coronary plaque stenosis should be considered. For this purpose, ^68^Ga-NODAGA-RGD PET/CT may aid our pathophysiological understanding of this important condition and help to identify patients at increased risk of adverse cardiovascular events.

Even if some previous literature reported on the role of RGD-based tracers in the imaging of atherosclerosis in humans, it needs to be further established. Thus far, only two recent studies have evaluated the imaging of atherosclerotic lesions with RGD-based PET agents in humans. Beer et al. documented the expression of αvβ3 integrin in macrophage infiltrates of plaque specimens obtained from a small sample of patients with high-grade carotid artery stenosis [12]. Jenkins et al. have demonstrated that in vivo expression of αvβ3 integrin with ^18^F-fluciclatide PET/CT in human aortic atheroma is associated with plaque burden and is increased in patients with recent myocardial infarction [13]. Our results are consistent with the promising results of these existing findings.

Regarding the colocalization of arterial uptake foci and calcification sites, the findings of this study add novel observations about RGD uptake in atherosclerotic lesions. In the present study, around 26% of ^68^Ga-NODAGA-RGD highest uptake foci were colocalized to calcification. Our study suggests that ^68^Ga-NODAGA-RGD accumulation particularly occurs mainly in the noncalcified vessel wall, which may indicate some association with pathophysiologic processes found in early atherosclerotic disease, whereas calcification is seen in more advanced lesions [20, 24].

When analyzing the intensity of ^68^Ga-NODAGA-RGD uptake in arterial segments, the measured TBRs were comparable with those for other established tracers used for plaque imaging. In the present study, the median intensity of tracer uptake as determined by TBR was 1.84 [1.62–2.04]. In a study using ^18^F-sodium fluoride for visualization of microcalcification in plaque, mean TBR was 2.3 ± 0.7 [34]. In another study using ^68^Ga-Pentixafor, mean TBR was 2.0 ± 0.5 [27]. In a study on vascular FDG uptake, mean TBR in the abdominal aorta was 1.57 ± 0.35 [18].

Holding promise as a marker of disease activity, targeting the integrin αvβ3 pathway could also represent an attractive therapeutic strategy in atherosclerosis. Humanized monoclonal antibodies against integrin αvβ3 (etaracizumab) have been developed [35]. And researchers develop attractive therapeutic strategies conjugating RGD to delivery systems (e.g., nanoparticles) to selectively deliver the chemotherapeutic agents to cells expressing integrin αvβ3 [36].

### Limitations

Because integrins αvβ3 are expressed in neovessels in plaques but also in CD68-positive macrophages, we are unable to ascertain whether ^68^Ga-NODAGA-RGD was binding preferentially to one or the other of these processes. However, non-selectivity between inflammation and angiogenesis is not necessarily a disadvantage. Both inflammation and angiogenesis are hallmark of unstable atheroma and to combine both processes could be of additional value. There was no histopathological correlation in our study to examine the relation between αvβ3 integrin expression by ^68^Ga-NODAGA-RGD uptake and plaque composition. One other limiting factor is the relatively lower spatial resolution of PET systems compared with other imaging techniques. Further developments in hybrid imaging promise to enhance the scope of molecular imaging. Although the use of ^68^Ga is widespread with ease of use and good availability of this radioisotope through a ^68^Ge/^68^Ga generator, the lower positron energy of ^18^F compared to ^68^Ga could potentially improve spatial resolution and reduce blurring effects. Given the difficulty in identifying the exact borders of the coronary arteries on the non-contrast-enhanced and non-gated CT image scans, we did not evaluate the coronary arteries. The two different PET camera systems have resulted in different imaging quality, but no significative difference was found between mean TBR values of patients who had a Discovery PET and of patients who had a Biograph Vision PET. The impact of previous anticancer therapies on vascular inflammation cannot be excluded (although none of the last previous systemic anticancer therapies included antiangiogenic drugs, and only six patients (7%) had systemic anticancer therapy within 4 weeks before imaging). Furthermore, given the relatively limited number of patients studied using univariate analyses, with a heterogeneous sample, we cannot exclude confounding of our results by other confounding factors.

## Conclusion

In conclusion, among consecutive patients who had been referred to our institution for a ^68^Ga-NODAGA-RGD PET/CT, ^68^Ga-NODAGA-RGD arterial uptake correlated with prior cardiovascular or cerebrovascular event and plaque burden and was increased in patients with previous clinically documented ASCVD. Although further study is required, our data suggest that RGD-based tracers hold promise as a non-invasive marker of disease activity in atherosclerosis, providing information on inflammation and angiogenesis, with clinical significance.

## Data Availability

The datasets used and/or analyzed during the current study are available from the corresponding author on reasonable request.
